# Aquatic Hyphomycetes from streams on Madeira Island (Portugal)

**DOI:** 10.3897/BDJ.8.e53690

**Published:** 2020-07-08

**Authors:** Pedro M. Raposeiro, Hélder Faustino, Verónica Ferreira, Vítor Gonçalves

**Affiliations:** 1 CIBIO, Research Center in Biodiversity and Genetic Resources, InBIO Associate Laboratory, Ponta Delgada, Portugal CIBIO, Research Center in Biodiversity and Genetic Resources, InBIO Associate Laboratory Ponta Delgada Portugal; 2 University of the Azores, Ponta Delgada, Portugal University of the Azores Ponta Delgada Portugal; 3 Faculty of Sciences and Technology, University of the Azores, Ponta Delgada, Portugal Faculty of Sciences and Technology, University of the Azores Ponta Delgada Portugal; 4 Universidade de Coimbra, MARE - Marine and Environmental Sciences Centre, Department of Life Sciences, Coimbra, Portugal Universidade de Coimbra, MARE - Marine and Environmental Sciences Centre, Department of Life Sciences Coimbra Portugal; 5 CIBIO, Research Center in Biodiversity and Genetic Resources, InBIO Associate Laboratory / Faculty of Sciences and Technology, University of the Azores, Ponta Delgada, Portugal CIBIO, Research Center in Biodiversity and Genetic Resources, InBIO Associate Laboratory / Faculty of Sciences and Technology, University of the Azores Ponta Delgada Portugal

**Keywords:** Ingoldian fungi, oceanic islands, freshwater enviroments, new records

## Abstract

**Background:**

Aquatic hyphomycetes are a phylogenetically heterogeneous group of fungi living preferentially in fast flowing, well-aerated forest streams. These fungi have worldwide distribution, but with the exception of *Articulospora tetracladia*, no aquatic hyphomycete taxon was previously recorded on Madeira Island. Aquatic hyphomycetes were sampled from 40 sites, distributed by 27 permanent streams in 2015, to provide the distribution of aquatic hyphomycetes in Madeira Island streams.

**New information:**

In this study, a total of 21 species of aquatic hyphomycetes were recorded belonging to three classes of Ascomycota. All taxa are new records for Madeira Archipelago, except *Articulospora tetracladia* and four are reported for the first time in Macaronesian biogeographic region.

## Introduction

Aquatic hyphomycetes, or Ingoldian fungi, are a phylogenetically heterogeneous group of fungi, composed mainly by the asexual stages of ascomycetes and basidiomycetes, living preferentially in fast flowing, well-aerated forest streams (Bärlocher 1992). Although aquatic fungi have been studied since the 1840s (Desmaziéres 1849), the knowledge of this fungal group is still scarce compared to their terrestrial counterparts. These fungi have worldwide distribution, but studies, so far, point to a higher species richness in temperate regions (Jones and Pang 2012, Duarte et al. 2016a, Seena et al. 2019). In fact, in temperate regions, they are the most important group of litter microbial decomposers in streams and rivers (Suberkropp and Klug 1974, Hieber and Gessner 2002, Gulis and Suberkropp 2003). Aquatic hyphomycetes play a fundamental role in the decomposition of plant litter of terrestrial origin, which is a key ecosystem process in forest streams that allows for the transfer of energy and nutrients to higher tropic levels, contributing to nutrient cycling (Wallace et al. 1997, Gessner et al. 2007, Gulis et al. 2019). Aquatic hyphomycetes colonise leaf litter soon after leaf immersion. They can promote litter mass loss directly by mineralising organic carbon and nutrients and by converting coarse into fine particulate organic matter (e.g. by the conidia production) (Gulis and Suberkropp 2003, Cornut et al. 2010) and indirectly by increasing litter palatability to shredders and facilitating physical fragmentation (Gulis et al. 2006, Graça and Cressa 2010). Aquatic hyphomycetes can be particularly important on oceanic island streams, where macroinvertebrate detritivores can be scarce (Benstead et al. 2009, Raposeiro et al. 2014, Ferreira et al. 2016b). In fact, fungal biomass, sporulation rates and litter decomposition by aquatic hyphomycetes in Atlantic islands was reported to be equivalent to those observed in temperate continental zones (Ferreira et al. 2016b, Ferreira et al. 2017). Despite their importance, little is known about aquatic hyphomycetes in oceanic island systems (e.g. Ranzoni 1979).

Interest in Madeiran terrestrial fungi started almost two centuries ago with the work of Holl (1830) that recorded a dozen species belonging to different groups. During the 20^th^ century, many mycological studies increased the number of records for Madeira Archipelago (North Atlantic), including numerous descriptions of species new to science (see Melo and Cardoso 2008 and references therein). According to Melo and Cardoso (2008), 743 fungal taxa were recorded for the Madeira Archipelago, with 99.3% occurring on Madeira Island. Despite their major relevance for the knowledge of Madeiran fungal biodiversity, these taxonomic studies focused on terrestrial ecosystems, whereas little is known about the aquatic habitat. The main objective of this paper is to provide the distribution of aquatic hyphomycetes in Madeira Island streams.

### Study area

**Figure 1.**
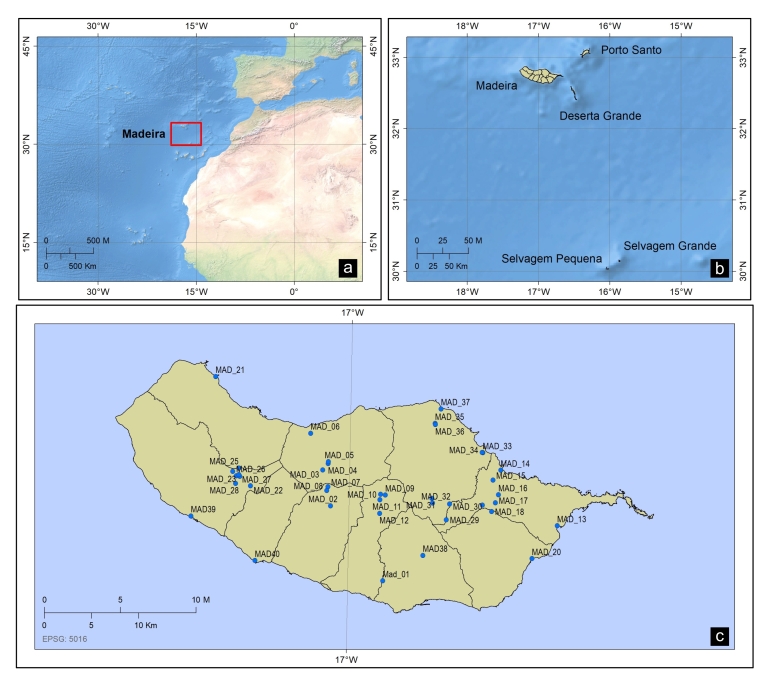
Geographical location of the study stream sites. **a.** Madeira Archipelago in the Atlantic Ocean highlighted by a square; **b.** Madeira Island in the Madeira Archipelago; **c.** Studied stream sites.

Madeira Island is located 600 km off the Atlantic coast of North Africa (Fig. 1). It has an area of 742 km^2^ and a maximum altitude of 1861 m (Pico Ruivo). Lying in the subtropical region, Madeira’s climate is influenced by winds from NE and the Canary Islands current. The Island has a mild oceanic climate, both in winter and summer with mild temperatures ranging from 15.9°C in February up to 22.3°C in August (average annual temperature of 18.7°C), relative humidity between 55 and 75% and annual rainfall between 500 and 1,000 mm (Santos et al. 2004).

Madeira Island comprises approximately 126 catchments and 200 streams (Marques 1994) ranging from 1st to 6th order. The radial drainage pattern of the watersheds is typical of oceanic islands as streams flow away from the island’s mountainous central peaks (Hughes 2006). Madeira stream drainage networks are typically narrow and short with very steep, shallow channels often characterised by turbulent, torrential and seasonal flow. Substrates are predominantly coarse, comprising bedrock, boulders, cobbles and sand. Due to complex orography and the altitudinal span of the Island, the vegetation and land use are distributed along the altitudinal gradient. Forested areas (native laurel forest and commercial plantations) and less impacted areas occupy the higher reaches of most catchments, while agricultural and urban land uses characterise more accessible middle and lower lying areas. Other observed impacts include organic pollution, nutrient enrichment via diffuse pollution and physical disturbance (bank reinforcement or modification in the riparian corridor).

## Materials and methods

Water columns were sampled for conidia of aquatic hyphomycetes from 40 sites (MAD1 – MAD40) distributed by 27 permanent streams (Suppl. material 1) in the spring of 2015. At each site, 5 litres of stream water were filtered through cellulose nitrate filters (47 mm diameter, 8 µm pore size; Whatman GF/C, GE Healthcare Europe GmbH, Little Chalfont, U.K.) using an electrical vacuum pump. It was connected to a rubber tube that collected water just below the stream surface. The filters were stained with cotton blue in 60% lactic acid (0.05%) and stored in individual Petri dishes isolated with Parafilm tape. In the laboratory, filters were cut in half, mounted on slides and scanned with a compound microscope (Leica DM2500, Leica Microsystems CMS GmbH, Wetzlar, Germany) at 200× magnification. Conidia were identified (based on the morphological characters) and counted following Gulis et al. (2005) and taxonomical classification was performed according to Index Fungorum. The taxonomic list, presented below, is available also in Darwin Core compliant format (see Suppl. materials 3, 4and Raposeiro et al. 2020).

## Checklists

### A checklist of Madeira aquatic hyphomycetes

#### Alatospora acuminata

Ingold, Trans. Br. mycol. Soc. 25 (4): 384 (1942)

3690A495-A84F-50C3-A2E6-2BEDD6529505

##### Distribution

Cosmopolitan (Duarte et al. 2016a, Seena et al. 2019).

##### Notes

Madeira distribution: Streams in agricultural and natural areas at low to moderate altitude: Ribeira de São Vicente (MAD04); Ribeira do Juncal (MAD15); Ribeira do Faial (MAD16); Ribeira Primeira (MAD18); Ribeira de São Jorge (MAD37).

Habitat: Submerged leaf litter [e.g. *Acer rubrum* L., *Alnus glutinosa* (L.) Gaertn., *Clethra arborea* Aiton, *Quercus robur* L., *Pittosporum undulatum* Vent., *Rhododendron maximum* L. (Gulis and Suberkropp 2003, Ferreira et al. 2006b, Ferreira et al. 2016b)].

#### Anguillospora crassa

Ingold, Trans. Br. mycol. Soc. 41 (3): 367 (1958)

98B6FBD1-05B6-5F95-9579-C0EC201B326E

##### Distribution

Cosmopolitan (Duarte et al. 2016a).

##### Notes

Madeira distribution: Streams in agricultural and natural areas at low to high altitude: Ribeira Brava (MAD08); Ribeira do Juncal (MAD15); Ribeira Primeira (MAD18); Ribeira do Alecrim (MAD22); Ribeira de São Roque do Faial (MAD33).

Habitat: Submerged leaf litter and wood veneers [e.g. *Acacia melanoxylon* R. Br., *Alnus glutinosa, Clethra arborea, Quercus robur, Ochroma pyramidale* (Cav.ex Lam.) Urb.- (Ferreira et al. 2006b, Ferreira et al. 2016b)].

#### Aquanectria submersa

(H.J. Huds.) L. Lombard & Crous, in Lombard, van der Merwe, Groenewald & Crous, Stud. Mycol. 80: 207 (2015)

672BA860-8D1E-5A9B-9963-0C35130DCB8E

##### Distribution

Cosmopolitan (Duarte et al. 2016a).

##### Notes

Madeira distribution: Stream in agricultural and natural areas at low to moderate altitude: Ribeira de São Vicente (MAD04); Ribeira Brava (MAD07); Ribeira do Juncal (MAD14, MAD15); Ribeira do Faial (MAD16); Ribeira da Janela (MAD21); Ribeira de São Roque do Faial (MAD33); Ribeira dos Arcos (MAD36); Ribeira de São Jorge (MAD17); Ribeira da Fonte do Bugio (MAD39).

Habitat: Submerged leaf litter [e.g. *Alnus glutinosa*, *Eucalyptus globulus* (Duarte et al. 2006, Gonçalves et al. 2007)]

#### Articulospora tetracladia

Ingold, Trans. Br. mycol. Soc. 25 (4): 376 (1942)

2C3D3687-6D98-56F5-B585-C09FC32D5F9F

##### Distribution

Cosmopolitan (Duarte et al. 2016a) and Madeira (Seena et al. 2012).

##### Notes

Madeira distribution: Streams in urban, agricultural and natural areas at low to high altitude: Ribeira dos Socorridos (MAD01, MAD09); Ribeira Brava (MAD02, MAD07, MAD08); Ribeira da Vargem (MAD03); Ribeira Grande (MAD05, MAD06); Ribeira da Gomeira (MAD10); Corgo da Ribeira de Anéis (MAD11); Ribeira do Juncal (MAD15); Ribeira do Faial (MAD16); Ribeira Primeira (MAD18); Ribeira do Machico (MAD19); Ribeira da Janela (MAD21, MAD24, MAD26, MAD27); Ribeira do Alecrim (MAD22, MAD28); Ribeira dos Cedros (MAD25); Ribeiro Frio (MAD29); Ribeira do Córrego do Arrochete (MAD30); Ribeira das Lajes (MAD32); Ribeira de São Roque do Faial (MAD33); Ribeira Seca (MAD34); Ribeira dos Arcos (MAD36); Ribeira de São Jorge (MAD37); Ribeira de Santa Luzia (MAD38).

Habitat: Submerged leaf litter [e.g. *Acer rubrum*, *Alnus glutinosa*, *Cryptomeria japonica* D. Don, *Ilex perado* Aiton, *Quercus robur*, *Pittosporum undulatum*, *Rhododendron maximum* (Gulis and Suberkropp 2003, Ferreira et al. 2006b, Ferreira et al. 2016b, Ferreira et al. 2017].

#### Campylospora chaetocladia

Ranzoni, Farlowia 4(3): 373 (1953)

0A79C7F8-B63C-58F2-A523-D57018E06C1B

##### Distribution

Cosmopolitan (Duarte et al. 2016a).

##### Notes

Madeira distribution: Streams in urban, agricultural and natural areas at low to high altitude: Ribeira Brava (MAD02, MAD08); Ribeira de São Vicente (MAD04); Ribeira Grande (MAD05, MAD06); Ribeira da Gomeira (MAD10); Ribeira do Machico (MAD13); Ribeira do Juncal (MAD15); Ribeira da Janela (MAD21, MAD26); Ribeira de São Roque do Faial (MAD33); Ribeira Seca (MAD34); Ribeira dos Arcos (MAD36); Ribeira de São Jorge (MAD37); Ribeira de Santa Luzia (MAD38); Ribeira da Ponta do Sol (MAD 40).

Habitat: Submerged leaf litter [e.g. *Acacia melanoxylon*, *Clethra arborea, Pittosporum undulatum* (Ferreira et al. 2016b)].

#### Clavariopsis aquatica

De Wild., Ann. Soc. Belge Microscop. 19: 201 (1895)

066670E2-4C6C-5FB6-8E48-69B0BCA62196

##### Distribution

Cosmopolitan (Duarte et al. 2016a, Seena et al. 2019).

##### Notes

Madeira distribution: Streams in urban, agricultural and natural areas at low to high altitude: Ribeira dos Socorridos (MAD01, MAD09); Ribeira Brava (MAD02, MAD07, MAD08); Ribeira da Vargem (MAD03); Ribeira de São Vicente (MAD04); Ribeira Grande (MAD05); Ribeira da Gomeira (MAD10); Corgo da Ribeira de Anéis (MAD11); Ribeira do Cidrão (MAD12); Ribeira do Juncal (MAD15); Ribeira do Faial (MAD16); Ribeira do Machico (MAD17); Ribeira da Janela (MAD21, MAD24, MAD26, MAD27); Ribeira dos Cedros (MAD25); Ribeira do Alecrim (MAD28); Ribeira do Córrego do Arrochete (MAD30); Ribeira da Metade (MAD31); Ribeira das Lajes (MAD32); Ribeira de São Roque do Faial (MAD33); Ribeira Seca (MAD34); Ribeira de São Jorge (MAD35, MAD37); Ribeira dos Arcos (MAD36); Ribeira de Santa Luzia (MAD38); Ribeira da Ponta do Sol (MAD 40).

Habitat: Submerged leaf litter and wood veneers [e.g. *Acacia melanoxylon*, *Alnus glutinosa*, *Clethra arborea*, *Cryptomeria japonica, Quercus robur, Ochroma pyramidale* (Ferreira et al. 2006b, Ferreira et al. 2016b)].

#### Clavatospora longibrachiata

(Ingold) Sv. Nilsson ex Marvanová & Sv. Nilsson, Trans. Br. mycol. Soc. 57 (3): 531 (1971)

9DC7849A-ABEE-5FF9-A9AF-B5E3A39416E1

##### Distribution

Cosmopolitan (Duarte et al. 2016a, Seena et al. 2019)

##### Notes

Madeira distribution: Streams in agricultural and natural areas at low to high altitude: Ribeira de São Vicente (MAD04); Ribeira Brava (MAD07); Ribeira dos Socorridos (MAD09); Ribeira do Machico (MAD17); Ribeira Primeira (MAD18); Ribeira do Alecrim (MAD22); Ribeira dos Cedros (MAD25); Ribeira da Janela (MAD26); Ribeira do Córrego do Arrochete (MAD30).

Habitat: Submerged leaf litter [e.g. *Alnus glutinosa*, *Clethra arborea*, *Quercus robur* (Ferreira et al. 2006a, Ferreira et al. 2016b)].

#### Fontanospora eccentrica

(R.H. Petersen) Dyko, Trans. Br. mycol. Soc. 70 (3): 412 (1978)

8A859C84-48D6-5382-BE6E-FC0C03AD9EDD

##### Distribution

Cosmopolitan (Duarte et al. 2016a).

##### Notes

Madeira distribution: Streams in natural areas at low to high altitude: Ribeira Brava (MAD07); Corgo da Ribeira de Anéis (MAD11); Ribeira do Córrego do Arrochete (MAD30); Ribeira de São Jorge (MAD37).

Habitat: Submerged leaf litter [e.g. *Ilex perado* (Ferreira et al. 2017)].

#### Geniculospora inflata

(Ingold) Sv. Nilsson ex Marvanová & Sv. Nilsson, Trans. Br. mycol. Soc. 57 (3): 532 (1971)

15B4E8EC-42DF-52E6-B357-42CBE90D2558

##### Distribution

Cosmopolitan (Duarte et al. 2016a).

##### Notes

Madeira distribution: Streams in natural areas at high altitude: Ribeira da Janela (MAD24, MAD26); Ribeira do Córrego do Arrochete (MAD30).

Habitat: Submerged leaf litter [e.g. *Alnus glutinosa* (Menéndez et al. 2012)].

#### Lemonniera aquatica

De Wild., Ann. Soc. Belge Microscop. 18: 147 (1894)

4EB4C881-2272-5B43-8610-CD169A244D18

##### Distribution

Cosmopolitan (Duarte et al. 2016a).

##### Notes

Madeira distribution: Streams in urban, agricultural and natural areas at low to high altitude: Ribeira dos Socorridos (MAD01, MAD09); Ribeira Grande (MAD05); Ribeira Brava (MAD07); Ribeira da Gomeira (MAD10); Ribeira do Juncal (MAD15); Ribeira do Faial (MAD16); Ribeira do Machico (MAD17, MAD19); Ribeira Primeira (MAD18); Ribeira da Janela (MAD24, MAD26, MAD27); Ribeira dos Cedros (MAD25); Ribeira do Alecrim (MAD28); Ribeira do Córrego do Arrochete (MAD30); Ribeira da Metade (MAD31); Ribeira das Lajes (MAD32); Ribeira dos Arcos (MAD36); Ribeira de São Jorge (MAD37); Ribeira de Santa Luzia (MAD38); Ribeira da Ponta do Sol (MAD 40).

Habitat: Submerged leaf litter and wood [e.g. *Acacia melanoxylon*, *Alnus glutinosa*, *Clethra arborea*, *Ilex perado, Ochroma pyramidale*, *Pittosporum undulatum* (Ferreira et al. 2006b, Ferreira et al. 2016b, Ferreira et al. 2017)].

#### Lemonniera sp.

De Wild., Ann. Soc. Belge Microscop. 18: 143 (1894)

0B03CE4C-1284-54D8-B683-C372DD43F676

##### Notes

Madeira distribution: Streams in natural areas at moderate altitude: Ribeira do Córrego do Arrochete (MAD30).

Habitat: Submerged leaf litter [e.g. *Ilex perado* (Ferreira et al. 2017)].

#### Lunulospora curvula

Ingold, Trans. Br. mycol. Soc. 25 (4): 209 (1942)

FE62BC1E-9920-5D72-B5CF-BB48EB6B146D

##### Distribution

Cosmopolitan (Duarte et al. 2016a, Seena et al. 2019).

##### Notes

Madeira distribution: Streams in urban, agricultural and natural areas at low to high altitude: Ribeira dos Socorridos (MAD01, MAD09); Ribeira Brava (MAD02, MAD07, MAD08); Ribeira da Vargem (MAD03); Ribeira de São Vicente (MAD04); Ribeira Grande (MAD05, MAD06); Ribeira da Gomeira (MAD10); Corgo da Ribeira de Anéis (MAD11); Ribeira do Cidrão (MAD12); Ribeira do Juncal (MAD14, MAD15); Ribeira do Faial (MAD16); Ribeira do Machico (MAD17); Ribeira Primeira (MAD18); Ribeira de Santa Cruz (MAD20); Ribeira da Janela (MAD21, MAD23, MAD24, MAD26); Ribeira dos Cedros (MAD25); Ribeira do Alecrim (MAD28); Ribeira da Metade (MAD31); Ribeira das Lajes (MAD32); Ribeira de São Roque do Faial (MAD33); Ribeira Seca (MAD34); Ribeira de São Jorge (MAD35, MAD37); Ribeira dos Arcos (MAD36); Ribeira de Santa Luzia (MAD38); Ribeira da Fonte do Bugio (MAD39); Ribeira da Ponta do Sol (MAD 40).

Habitat: Submerged leaf litter [e.g. *Acer rubrum*, *Alnus glutinosa*, *Eucalyptus globulus*, *Quercus robur*, *Rhododendron maximum* (Gulis and Suberkropp 2003, Ferreira et al. 2006b, Gonçalves et al. 2007)].

#### Mycofalcella calcarata

Marvanová, Om-Kalth. & J. Webster, Nova Hedwigia 56 (3-4): 402 (1993)

2C7206C5-41F1-55B0-81DD-50EACEDD8BE9

##### Distribution

Cosmopolitian (Sales et al. 2015, Duarte et al. 2016a).

##### Notes

Madeira distribution: Streams in agricultural and natural areas at moderate altitude: Ribeira Brava (MAD02, MAD07, MAD08); Ribeira de São Vicente (MAD04); Ribeira Primeira (MAD18); Ribeiro Frio (MAD29).

Habitat: Submerged leaf litter [e.g. *Alnus glutinosa*, *Pittosporum undulatum, Quercus robur* (Cornut et al. 2010, Duarte et al. 2016b, Ferreira et al. 2016b)].

#### Neonectria lugdunensis

(Sacc. & Therry) L. Lombard & Crous, in Lombard, van der Merwe, Groenewald & Crous, Phytopath. Mediterr. 53(3): 528 (2014)

9B3C3176-C801-5961-B75C-48C31DFB8EED

##### Distribution

Cosmopolitan (Duarte et al. 2016a).

##### Notes

Madeira distribution: Streams in urban, agricultural and natural areas at low to high altitude: Ribeira Brava (MAD02); Ribeira da Vargem (MAD03); Ribeira Brava (MAD07, MAD08); Ribeira dos Socorridos (MAD09); Ribeira do Juncal (MAD14, MAD15); Ribeira do Machico (MAD17); Ribeira Primeira (MAD18); Ribeira da Janela (MAD26, MAD27); Ribeira do Alecrim (MAD28); Ribeira do Córrego do Arrochete (MAD30).

Habitat: Submerged leaf litter [e.g. *Acacia melanoxylon*, *Acer rubrum*, *Clethra arborea*, *Cryptomeria japonica*, *Eucalyptus globulus* Labill., *Ilex perado, Pittosporum undulatum*, *Rhododendron maximum* (Gulis and Suberkropp 2003, Ferreira et al. 2006b, Gonçalves et al. 2007, Ferreira et al. 2016a, Ferreira et al. 2017)].

#### Tetrachaetum elegans

Ingold, Trans. Br. mycol. Soc. 25 (4): 381 (1942)

66C0859A-B534-569F-91A7-1B86F5386203

##### Distribution

Cosmopolitan (Duarte et al. 2016a).

##### Notes

Madeira distribution: Streams in urban, agricultural and natural areas at low to high altitude: Ribeira dos Socorridos (MAD01, MAD09); Ribeira de São Vicente (MAD04); Ribeira Brava (MAD07, MAD08); Ribeira do Cidrão (MAD12); Ribeira do Faial (MAD16); Ribeira do Machico (MAD17, MAD19); Ribeira Primeira (MAD18); Ribeira da Janela (MAD24, MAD26, MAD27); Ribeira dos Cedros (MAD25); Ribeira do Alecrim (MAD28); Ribeira do Córrego do Arrochete (MAD30); Ribeira da Metade (MAD31); Ribeira das Lajes (MAD32); Ribeira Seca (MAD34); Ribeira de Santa Luzia (MAD38).

Habitat: Submerged leaf litter [e.g. *Acacia melanoxylon*, *Acer rubrum*, *Alnus glutinosa*, *Clethra arborea*, *Cryptomeria japonica*, *Eucalyptus globulus*, *Ilex perado, Pittosporum undulatum*, *Quercus robur*, *Rhododendron maximum* (Gulis and Suberkropp 2003, Ferreira et al. 2006b, Gonçalves et al. 2007, Ferreira et al. 2016b, Ferreira et al. 2017)].

#### Tetracladium furcatum

Descals, Trans. Br. mycol. Soc. 80 (1): 70 (1983)

4A01C23D-7A26-5A8C-AB8D-A5DA171E8D13

##### Distribution

Cosmopolitan (Duarte et al. 2016a).

##### Notes

Madeira distribution: Streams in agricultural areas at low altitude: Ribeira dos Socorridos (MAD01); Ribeira de Santa Luzia (MAD38).

Habitat: Submerged leaf litter [e.g. *Alnus glutinosa* (Pascoal et al. 2005)].

#### Tetracladium marchalianum

De Wild., Ann. Soc. Belge Microscop. 17: 39 (1893)

4C379B98-63BA-517A-92C0-C484B1A790AC

##### Distribution

Cosmopolitan (Duarte et al. 2016a).

##### Notes

Madeira distribution: Streams in urban, agricultural and natural areas at low to high altitude: Ribeira dos Socorridos (MAD01, MAD09); Ribeira Brava (MAD02, MAD07, MAD08); Ribeira da Vargem (MAD03); Ribeira de São Vicente (MAD04); Ribeira Grande (MAD05, MAD06); Ribeira da Gomeira (MAD10); Corgo da Ribeira de Anéis (MAD11); Ribeira do Cidrão (MAD12); Ribeira do Juncal (MAD14, MAD15); Ribeira do Alecrim (MAD22); Ribeira da Janela (MAD24, MAD26); Ribeira do Córrego do Arrochete (MAD30); Ribeira da Metade (MAD31); Ribeira das Lajes (MAD32); Ribeira de São Roque do Faial (MAD33); Ribeira Seca (MAD34); Ribeira dos Arcos (MAD36); Ribeira de São Jorge (MAD37); Ribeira de Santa Luzia (MAD38); Ribeira da Fonte do Bugio (MAD39); Ribeira da Ponta do Sol (MAD 40).

Habitat: Submerged leaf litter [e.g. *Acacia melanoxylon*, *Eucalyptus globulus*, *Ilex perado, Pittosporum undulatum* (Gonçalves et al. 2007, Ferreira et al. 2016b, Ferreira et al. 2017)].

#### Tetracladium setigerum

(Grove) Ingold, Trans. Br. mycol. Soc. 25(4): 371(1942)

29EA0E24-82EE-5239-85A5-1555AED4BAD6

##### Distribution

Cosmopolitan (Duarte et al. 2016a).

##### Notes

Madeira distribution: Streams in agricultural areas at low to moderate altitude: Ribeira dos Socorridos (MAD01); Ribeira Brava (MAD02); Ribeira do Juncal (MAD15).

Habitat: Submerged leaf litter [e.g. *Clethra arborea*, *Ilex perado* (Ferreira et al. 2016b, Ferreira et al. 2017)].

#### Tricladium chaetocladium

Ingold, Trans. Br. mycol. Soc. 63(3): 624 (1974)

68D79614-DC15-5AF0-991B-F21A84FD1786

##### Distribution

Cosmopolitan (Duarte et al. 2016a).

##### Notes

Madeira distribution: Streams in urban, agricultural and natural areas at low to high altitude: Ribeira dos Socorridos (MAD01); Ribeira de São Vicente (MAD04); Ribeira Grande (MAD05); Ribeira Brava (MAD07, MAD08); Ribeira do Cidrão (MAD12); Ribeira do Juncal (MAD14, MAD15); Ribeira do Faial (MAD16); Ribeira do Machico (MAD17); Ribeira Primeira (MAD18, MAD19); Ribeira da Janela (MAD24, MAD26); Ribeiro Frio (MAD29); Ribeira do Córrego do Arrochete (MAD30); Ribeira da Metade (MAD31); Ribeira das Lajes (MAD32); Ribeira de São Jorge (MAD35); Ribeira dos Arcos (MAD36).

Habitat: Submerged leaf litter [e.g. *Acacia melanoxylon*, *Acer rubrum*, *Alnus glutinosa*, *Clethra arborea*, *Cryptomeria japonica*, *Ilex perado, Pittosporum undulatum*, *Quercus robur*, *Rhododendron maximum* (Gulis and Suberkropp 2003, Ferreira et al. 2006b, Ferreira et al. 2016b, Ferreira et al. 2017)].

#### Triscelophorus acuminatus

Nawawi, Trans. Br. mycol. Soc. 64 (2): 346 (1975)

F45FE042-099D-5651-8E96-FC87B0CB74C9

##### Distribution

Cosmopolitan (Duarte et al. 2016a).

##### Notes

Madeira distribution: Streams in agricultural and natural areas at low to moderate altitude: Ribeira dos Socorridos (MAD01); Ribeira Brava (MAD08); Ribeira do Juncal (MAD15); Ribeira do Faial (MAD16); Ribeira do Machico (MAD17); Ribeira Primeira (MAD18); Ribeira da Janela (MAD21); Ribeiro Frio (MAD29); Ribeira do Córrego do Arrochete (MAD30); Ribeira das Lajes (MAD32); Ribeira Seca (MAD34); Ribeira de São Jorge (MAD35); Ribeira dos Arcos (MAD36); Ribeira de Santa Luzia (MAD38).

Habitat: Submerged leaf litter [e.g. *Acacia melanoxylon*, *Alnus glutinosa*, *Clethra arborea*, *Ilex perado, Pittosporum undulatum*, *Quercus robur* (Ferreira et al. 2006b, Ferreira et al. 2016b, Ferreira et al. 2017].

#### Triscelophorus monosporus

Ingold, Trans. Br. mycol. Soc. 26(3-4): 152 (1943)

E9A99FEC-221E-5C74-98E4-1A77CEA78B80

##### Distribution

Cosmopolitan (Duarte et al. 2016a).

##### Notes

Madeira distribution: Streams in urban, agricultural and natural areas at low to high altitude: Ribeira dos Socorridos (MAD01); Ribeira Brava (MAD02, MAD07, MAD08); Ribeira de São Vicente (MAD04); Ribeira Grande (MAD05, MAD06); Ribeira da Gomeira (MAD10); Ribeira do Cidrão (MAD12); Ribeira do Juncal (MAD14, MAD15); Ribeira do Faial (MAD16); Ribeira do Machico (MAD17); Ribeira Primeira (MAD18); Ribeira da Janela (MAD21); Ribeira dos Cedros (MAD25); Ribeiro Frio (MAD29); Ribeira de São Roque do Faial (MAD33); Ribeira Seca (MAD34); Ribeira de São Jorge (MAD35, MAD37); Ribeira dos Arcos (MAD36); Ribeira de Santa Luzia (MAD38).

Habitat: Submerged leaf litter [e.g. *Acacia melanoxylon*, *Acer rubrum* L., *Alnus glutinosa*, *Clethra arborea*, *Eucalyptus globulus*, *Ilex perado, Pittosporum undulatum*, *Quercus robur*, *Rhododendron maximum* (Gulis and Suberkropp 2003, Ferreira et al. 2006a, Ferreira et al. 2006b, Gonçalves et al. 2007, Ferreira et al. 2016b, Ferreira et al. 2017)].

## Analysis

In the present study, we found a total of 21 aquatic hyphomycetes species, representing 17 genera in the phylum Ascomycota (Suppl. material 2). Amongst the fungal classes found in our study (Leotiomycetes, Sordariomycetes and Dothideomycetes), Leotiomycetes encompassed 43% of the taxa recorded. At the order level, the 21 ascomycetes were distributed by Helotiales (12 spp.), Hypocreales (2 spp.), Leotiales (1 sp.), Microascales (1 sp.), Sordariales (1 sp.) and Incertae sedis (4 spp.), according to Anderson and Marvanová 2020, Wijayawardene et al. 2020.

From the 21 species identified, none occurred in all 40 studied stream sites and only 8 taxa occurred in more than 50% of the stream sites: *Articulospora tetracladia*, *Clavariopsis aquatica*, *Lemonniera aquatica*, *Lunulospora curvula*, *Tetrachaetum elegans, Tetracladium marchalianum*, *Tricladium chaetocladium* and *Triscelophorus monosporus*, which were the most ubiquitous aquatic hyphomycetes in Madeira streams. Two taxa, *Lemonniera* sp. and *Tetracladium furcatum*, had a sporadic occurrence, being found at only one or two sampling sites. A maximum of 14 species was recorded in MAD15 and a minimum of one species in MAD13 and MAD20, with a mean richness of eight species per stream site. Higher altitude stream sites (> 800 m a.s.l.) in natural areas, such as MAD07, MAD08, MAD24 and MAD26, displayed higher taxa richness (11.0 ± 0.9, mean ± SE), compared with coastal (< 25 m a.s.l.) and urban stream sites, such as MAD13, MAD20, MAD32, MAD33, MAD37, MAD38, MAD39 and MAD40 (5.8 ± 1.2). All species, with the exception of *Articulospora tetracladia* reported by Seena et al. (2012), were new records for Madeira Archipelago and the following section provides brief notes on the records with information on their wider distribution patterns and habitat.

## Discussion

Here we present the first study that explored the distribution of aquatic hyphomycetes in insular streams from Madeira Island. Twenty-one taxa are recorded for Madeira Island, which is lower than what is reported for the Azores archipelago (41 species; see Ferreira et al. 2016b, Ferreira et al. 2017, Balibrea et al. 2020). However, these numbers cannot be used to draw conclusions about aquatic hyphomycetes species richness in each archipelago since sampling has used different approaches and has been limited in both archipelagos: in Madeira, aquatic hyphomycetes were sampled on one occasion from water in a large number of streams spatially distributed to cover the entire island surface, while in the Azores, aquatic hyphomycetes have been sampled on multiple occasions from submerged litter in few streams in one of the nine islands (São Miguel) of the Archipelago. In this context, it is essential to increase the sampling effort for both archipelagos, as well as to survey multiple matrices (water, foam, different decomposing litter species and types) in order to find a greater diversity of aquatic hyphomycetes. To the best of our knowledge, no data of recorded species exist for the other Macaronesia archipelagos, such as Canary Islands and Cabo Verde Archipelago.

The aquatic hyphomycete assemblages of Madeira were composed mainly by ascomycetes with a cosmopolitan distribution (Duarte et al. 2016a, Seena et al. 2019) which are also known from other oceanic islands (Ranzoni 1979, Ferreira et al. 2016b, Ferreira et al. 2017,Balibrea et al. 2020). In fact, other studies (Fenchel 1993, Finlay and Clarke 1999, Finlay 2002, Finlay and Fenchel 2004) suggested the high capacity of dispersal of microorganisms with few geographical barriers when compared to macroorganisms, such as freshwater macroinvertebrates (Hughes et al. 1998, Hughes and Malmqvist 2005, Raposeiro et al. 2012). While at the global scale, most of the aquatic hyphomycetes species have a cosmopolitan distribution (although some level of endemism was observed in some studies; see Duarte et al. 2016a, Seena et al. 2019), at a local scale, their assemblages are strongly influenced by environmental factors that dominate over the spatial processes (Barlocher and Graça 2002, Gulis and Suberkropp 2003, Rajashekhar and Kaveriappa 2003, Heino et al. 2004, Ferreira et al. 2006a, Cornut et al. 2012, Ferreira et al. 2016a, Duarte et al. 2017), which can explain the differences in the distribution of aquatic hyphomycetes species observed in Madeira streams. This is in line with the hypothesis of Baas-Becking (1934), which claims that “everything is everywhere”, but microbial assemblages are controlled by environmental factors. However, we must have in mind that the actual knowledge on the global distribution of aquatic hyphomycetes is biased to certain geographical areas where the sampling efforts have been concentrated (Duarte et al. 2016a).

To better understand the complexity of these unique insular streams, further research on taxonomy, population dynamics, litter decomposition, sensitivity to environmental conditions, amongst others, need to be carried out. Additionally, replication and larger datasets are required to better understand insular aquatic hyphomycete communities and how they response to environmental changes.

## Supplementary Material

F5E8BBEA-3734-5ED5-97BF-94E886525345 10.3897/BDJ.8.e53690.suppl1 Supplementary material 1 Sampling codes and location of the 40 studied stream sites on Madeira island Data type Table File: oo_416221.csv https://binary.pensoft.net/file/416221 Raposeiro, P.M.; Faustino, H.; Ferreira, V.; Gonçalves, V.

705C15EF-9C4A-59AB-A266-8F6D8A578CC7 10.3897/BDJ.8.e53690.suppl2 Supplementary material 2 Presence of aquatic hyphomycetes taxa in 40 Madeiran stream sites and total taxa richness of each stream site Data type Occurences Brief description Record of quatic hyphomycetes taxa in 40 Madeiran stream sites and total taxa richness of each stream site File: oo_403980.csv https://binary.pensoft.net/file/403980 Raposeiro, P.M.; Faustino, H.; Ferreira, V.; Gonçalves, V.

BA4C32BD-206F-5130-8D8C-D6AD1CA6519E 10.3897/BDJ.8.e53690.suppl3 Supplementary material 3 Aquatic Hyphomycetes from insular streams (Madeira, Portugal) Data type Data records Brief description Metafile File: oo_404009.xml https://binary.pensoft.net/file/404009 Raposeiro, P.M.; Faustino, H.; Ferreira, V.; Gonçalves, V.

23207FAB-3574-536A-BFDF-036E130E693C 10.3897/BDJ.8.e53690.suppl4 Supplementary material 4 Occurrence of the aquatic hyphomycete species found in this study in 15 geographic regions defined based on the geographic location and climatic influence in the western and eastern hemispheres (adapted from Duarte et al. 2016) Data type Table Brief description Table File: oo_420217.csv https://binary.pensoft.net/file/420217 Raposeiro, P.M.; Faustino, H.; Ferreira, V.; Gonçalves, V.

XML Treatment for Alatospora acuminata

XML Treatment for Anguillospora crassa

XML Treatment for Aquanectria submersa

XML Treatment for Articulospora tetracladia

XML Treatment for Campylospora chaetocladia

XML Treatment for Clavariopsis aquatica

XML Treatment for Clavatospora longibrachiata

XML Treatment for Fontanospora eccentrica

XML Treatment for Geniculospora inflata

XML Treatment for Lemonniera aquatica

XML Treatment for Lemonniera sp.

XML Treatment for Lunulospora curvula

XML Treatment for Mycofalcella calcarata

XML Treatment for Neonectria lugdunensis

XML Treatment for Tetrachaetum elegans

XML Treatment for Tetracladium furcatum

XML Treatment for Tetracladium marchalianum

XML Treatment for Tetracladium setigerum

XML Treatment for Tricladium chaetocladium

XML Treatment for Triscelophorus acuminatus

XML Treatment for Triscelophorus monosporus
